# Inhibition of Lipid Oxidation Increases Glucose Metabolism and Enhances 2-Deoxy-2-[^18^F]Fluoro-d-Glucose Uptake in Prostate Cancer Mouse Xenografts

**DOI:** 10.1007/s11307-014-0814-4

**Published:** 2015-01-06

**Authors:** Isabel R. Schlaepfer, L. Michael Glodé, Carolyn A. Hitz, Colton T. Pac, Kristen E. Boyle, Paul Maroni, Gagan Deep, Rajesh Agarwal, Scott M. Lucia, Scott D. Cramer, Natalie J. Serkova, Robert H. Eckel

**Affiliations:** 1 Division of Medical Oncology, Genitourinary Cancer Program, University of Colorado School of Medicine, MS 8117 12801 E. 17th Ave, Room L18-8101D, Aurora, CO 80045 USA; 2 Division of Endocrinology Metabolism and Diabetes, University of Colorado Denver, Aurora, CO 80045 USA; 3 Department of Pharmacology, University of Colorado Denver, Aurora, CO 80045 USA; 4 Department of Pediatrics, University of Colorado Denver, Aurora, CO 80045 USA; 5 Department of Surgery, University of Colorado Denver, Aurora, CO 80045 USA; 6 School of Pharmacy, University of Colorado Denver, Aurora, CO 80045 USA; 7 Department of Pathology, University of Colorado Denver, Aurora, CO 80045 USA; 8 Department of Anesthesiology, University of Colorado Denver, Aurora, CO 80045 USA

**Keywords:** CPT1A, Etomoxir, FDG-PET, Glycolysis, Fatty acid, Beta-oxidation, Mitochondria

## Abstract

**Purpose:**

Prostate cancer (PCa) is the second most common cause of cancer-related death among men in the United States. Due to the lipid-driven metabolic phenotype of PCa, imaging with 2-deoxy-2-[^18^F]fluoro-d-glucose ([^18^F]FDG) is suboptimal, since tumors tend to have low avidity for glucose.

**Procedures:**

We have used the fat oxidation inhibitor etomoxir (2-[6-(4-chlorophenoxy)-hexyl]oxirane-2-carboxylate) that targets carnitine-palmitoyl-transferase-1 (CPT-1) to increase glucose uptake in PCa cell lines. Small hairpin RNA specific for CPT1A was used to confirm the glycolytic switch induced by etomoxir *in vitro*. Systemic etomoxir treatment was used to enhance [^18^F]FDG-positron emission tomography ([^18^F]FDG-PET) imaging in PCa xenograft mouse models in 24 h.

**Results:**

PCa cells significantly oxidize more of circulating fatty acids than benign cells via CPT-1 enzyme, and blocking this lipid oxidation resulted in activation of the Warburg effect and enhanced [^18^F]FDG signal in PCa mouse models.

**Conclusions:**

Inhibition of lipid oxidation plays a major role in elevating glucose metabolism of PCa cells, with potential for imaging enhancement that could also be extended to other cancers.

**Electronic supplementary material:**

The online version of this article (doi:10.1007/s11307-014-0814-4) contains supplementary material, which is available to authorized users.

## Introduction

Prostate cancer is the most common malignancy in men and the second leading cause of cancer in the USA. The interplay of genetic and metabolic events that lead to prostate cancer remain unclear, but recent studies increasingly recognize the role of highly elevated lipid metabolism in prostate cancer (PCa) research [1]. The metabolism of PCa is unique in comparison to other cancers because it is not dependent on the strong aerobic glycolysis typical of solid tumors, also known as the Warburg effect. In contrast, PCa cells preferentially shunt glucose carbons towards lipogenesis [2] and rely more on beta-oxidation to satisfy their energetic needs [3]. This reduced dependence on glucose is likely the basis of a decreased positron emission tomography (PET) signal with 2-deoxy-2-[^18^F]fluoro-d-glucose ([^18^F]FDG) tracers, especially in localized tumors [4]. However, the ability of PCa tumors to perform aerobic glycolysis remains intact, as new magnetic resonance spectroscopic imaging techniques indicate that C-13 hyperpolarized pyruvate delivered to mouse prostate or PCa patients is readily transformed to lactate [5, 6].

The exact mechanisms of glucose uptake and lipid metabolism in PCa cells remain unknown. Increased glucose uptake is only observed in advanced metastatic PCa, while lipid turnover can be observed in both primary and advanced PCa, likely accounting for the differences in detection of prostate cancers by PET-glucose imaging techniques [7]. In fact, studies have shown that [^18^F]FDG is readily taken up by cultured metastatic PCa cells and this uptake is strongly increased with hypoxia, a known activator of glycolysis in cancer cells [8].

Clinically safe lipid oxidation inhibitors such as etomoxir could be used to alter lipid metabolism and aerobic glycolysis in PCa cells. Etomoxir is an irreversible inhibitor of the long-chain fatty acid transporter and has been used in the treatment of heart failure as a metabolic inducer for the glycolytic switch [9]. It works by inhibiting carnitine-palmitoyl-transferase-1 (CPT-1), an enzyme anchored in the outer membrane of the mitochondria. Etomoxir blocks the entry of long-chain fatty acids into the mitochondria for oxidation, forcing cells to utilize oxidation of glucose for energy.

Blocking CPT-1 activity with etomoxir produces therapeutic benefits in the treatment of heart failure by shifting the energy supply from fatty acids to glucose [10]. Etomoxir also shows this therapeutic effect in PCa tumors [11], suggesting that the metabolic switch between lipids and glucose is also operating in PCa cells [12]. We hypothesized that this shift towards glucose could be also happening in lipid-utilizing cells (like PCa cells) to increase radio-labeled glucose uptake and enhance [^18^F]FDG uptake *in vivo*. In this study, we examined the metabolic effects of blocking lipid oxidation in PCa cells *in vitro* as well as *in vivo*, using mouse xenograft models and the transgenic adenocarcinoma of mouse prostate (TRAMP) model for [^18^F]FDG-PET imaging analysis.

## Materials and Methods

### Cell Culture and Drug Treatments

Cells were obtained from the University of Colorado Cancer Center and were authenticated by Single Tandem Repeat analysis (University of Colorado Cancer Center). Cells were passaged in RPMI (except VCaP cells in DMEM) (Invitrogen) containing 5 % fetal bovine Serum supplemented with amino acids and insulin (Hyclone). Human primary prostate-derived cells were isolated (IRB protocol: 00–812) and expanded in keratinocyte medium as previously described [13]. PC3-LUC cells were from Applied Biological Materials ABM and grown in Prigrow IV media (Applied Biological Materials ABM-TM004) plus 10 % FBS and 1 % P/S. Etomoxir-HCl (Sigma) was dissolved into a 10 mM stock solution in water and stored at −20 °C.

### Lipid Oxidation

Cells were plated in 12-well plates and grown to 70 % confluence in their respective growth media conditions and with 150 uM etomoxir at the indicated times. At the time of the assay, 1 mM carnitine, 100 uM bovine serum albumin (BSA)-conjugated fatty acids (Sigma) and C-14 labeled fatty acids (1 uCi/ml; PerkinElmer) and fresh media were added to the cells for 3 h. Entrapment of the generated [^14^C]CO_2_ was done by transferring the media to a sealed dual-chamber plate and injecting percloric acid to release the ^14^CO2, which was trapped in a NaOH solution in the adjacent well as previously described [14]. The radioactivity in the NaOH solution (containing the trapped [^14^C]CO_2_) was measured by scintillation counting (Beckman). Cells were washed twice with PBS, harvested in 200 ul of 0.05 % SDS lysis buffer and stored at −80 ° C for subsequent determination of protein concentration.

### Glucose Uptake

Basal glucose uptakes were determined as previously reported for human cells *in vitro* [15]. Briefly, cells (10^5^) in six-well plates were incubated in Krebs–Ringer bicarbonate-HEPES (KRBH) buffer (130 mM NaCl, 5 mM KCl, 1.3 mM CaCl_2_, 1.3 mM MgSO_4_, 25 mM HEPES, pH 7.4) containing 1 % fatty acid-free BSA (Sigma-Aldrich), 2-deoxy-d-glucose (0.5 mM; Sigma-Aldrich), and 2-deoxy-d-[1,2-^3^H]glucose (GE Healthcare) for 20 min at 37 °C. The cells were washed with ice-cold KRBH buffer, harvested in 800 μl of lysis buffer (Pierce Biotechnology) and then added to scintillation vials containing 5 ml of scintillation fluid. Radioactive counts were determined with a scintillation counter (LS6500; Beckman Coulter). Counts were converted to moles of glucose taken up and normalized to the protein concentration of the lysates.

### NMR Analysis of 1-[^13^C]Glucose Uptake and 3-[^13^C]-actate Export *In Vitro*

VCaP cells were incubated with 5 mM of 1-[^13^C]glucose (Cambridge Isotope Laboratories) and with vehicle or etomoxir drug for 24 h. Incubation media (9 ml) were collected and lyophilized overnight. Lyophilized media were re-dissolved in 0.5 ml of deuterium oxide, and the samples were analyzed by quantitative ^1^H- and ^13^C-nuclear magnetic resonance (NMR) spectroscopy [16]

### RT-PCR and shRNA Constructs Targeting CPT1A

cDNA was amplified by real-time polymerase chain reaction (PCR) using SYBR green (Applied Biosystems) detection. Specific primers are listed in Electronic supplementary material (ESM) Table 1. TRCN0000036279 and TRCN0000036281 CPT1A small hairpin RNAs (shRNAs) s and the non-targeting control SHC002 were purchased from Functional Genomics Core, Boulder, Colorado. Lentiviral transduction and selection were performed according to Sigma's MISSION protocol but using lentiviral packing plasmids pCMV-R8.74psPAX2 and VSV-G/pMD2.G and transfection reagent TransIT-LT1 (Mirius) as described [11].

### Immunoblots

Protein extracts (20 μg) were separated on a 7.5 % SDS-PAGE gel and transferred to nitrocellulose (Invitrogen) membranes for Western blot analysis. All antibodies were from Cell-Signaling. Band signals were visualized with ECL (Pierce).

### Xenograft Production and TRAMP Mice

Male athymic nude mice, 4–6 weeks old (Harlan Laboratories), were housed in the University of Colorado Center for Comparative Medicine. Subcutaneous xenografts were generated by injecting human VCaP cells in the flank of mice as described [11]. Approximately, 2 million cells were used for each injection. When tumors were palpable they were used for scans. Treatment was carried out with a single intraperitoneal injection of etomoxir (20 mg/kg) immediately after the basal PET scan. After 24 h the mice were subjected to a second PET scan. Mice were euthanized when no longer radioactive. For orthotopic xenografts, PC3-LUC cells (PC3 cells expressing the luciferase reporter) were injected in the right anterior lobe of prostates and monitored by bioluminescence with intraperitoneal injection of luciferin (IVIS imaging systems, Perkin Elmer). TRAMP transgenic mice were purchased from JAX® Mice at 6–8 weeks of age. All procedures were carried out under an approved University of Colorado Animal Care protocol.

### [^18^F]FDG-PET, Magnetic Resonance Imaging

Single-position, whole-body imaging was performed using a Siemens Inveon PET scanner at the University of Colorado Cancer Center Animal Imaging Core. FDG-PET was performed before treatment (basal) and after 24 h of systemic etomoxir (20 mg/kg) treatment. Mice were fasted for 6 h before [^18^F]FDG injection, and blood glucose levels was assessed prior to the [^18^F]FDG injection (all animals had <80 mg/ml of circulating glucose). Approximately 250 μCi of [^18^F]FDG, obtained through University of Colorado Hospital (PetNet solutions), was administered by tail vein injection to conscious animals (the precise dose was assessed by measuring the syringe before and after the injection) [17]. Animals were maintained in temperature-controlled cages for 1 h to allow for [^18^F]FDG uptake in tumors. Under isoflurane anesthesia (2.5 %), animals were placed on a warm pad (m2m Imaging), and a 10-min emission scan was acquired. [^18^F]FDG uptake in mouse tumor xenografts, orthototpic tumors, as well as control tissues (prostate, muscle, heart, and brown fat) was done by analyzing the micro-PET images with the ASIProVM (Concorde Microsystems) software. Regions of interest were drawn with the trace command around the tumors on scan slices, and the total activity of all tumor slices was summed. Total activity was divided by the time-corrected dose-delivered [time-corrected dose = dose injected × exp(−0.006317 *x t*)], where *t* is the time between the injection and scan time, and it is shown as the fold change of the baseline scan of each respective tumor. Representative images were generated using the Siemens Inveon Research Workplace software (v3.1.2). Conventional proton-density weighted MRI was used to assess TRAMP prostate size growth as described [18].

#### Statistical Analysis

Analysis was done with SPSSv21 (IBM) using *t* tests and ANOVA Tukey’s *post hoc* analysis. *P* < 0.05 was considered significant. All tests were two-sided. Data represent mean ± SD.

## Results

### CPT1A-Mediated Blockade of Beta-Oxidation Increases Glucose Uptake in Prostate Cells

**Fig. 1:**
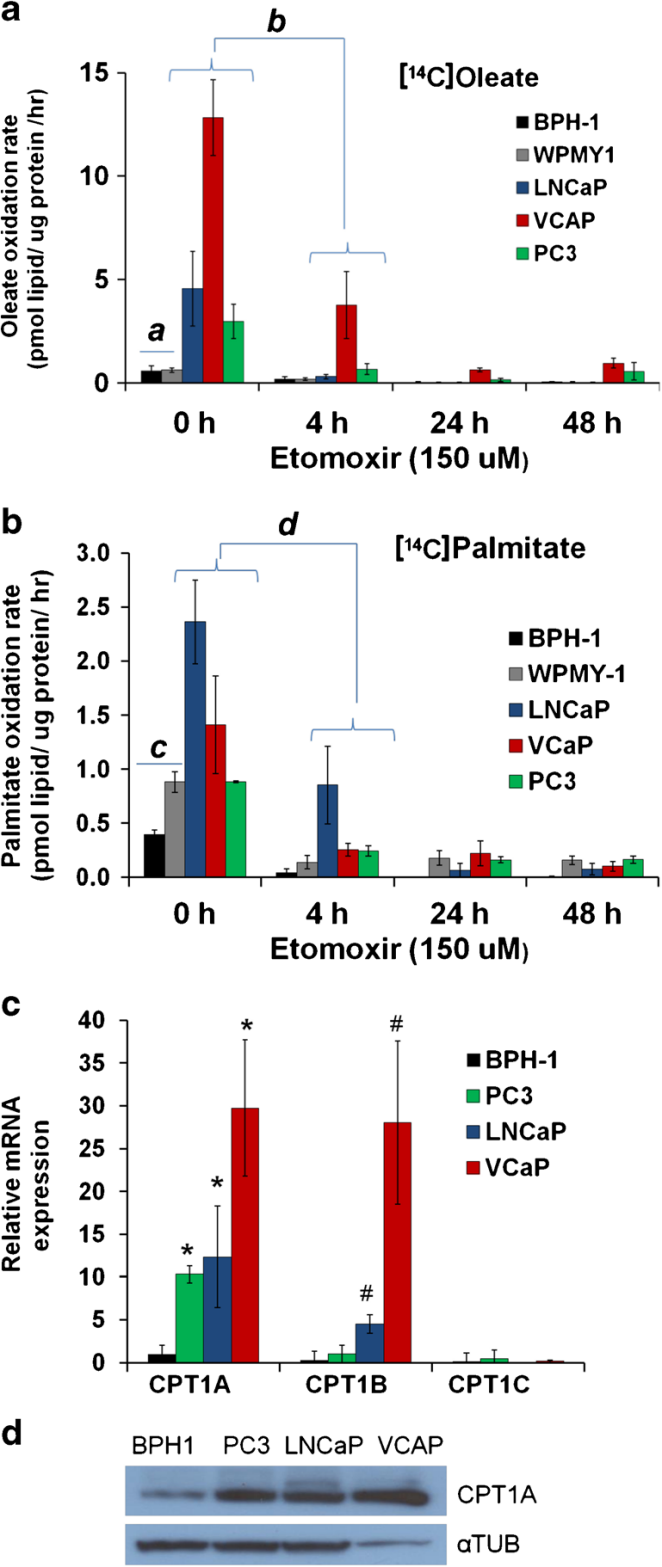
Lipid oxidation is abundant in prostate cancer cells and interrupted by etomoxir. **a**) The effect of etomoxir (150 μM) on the [^14^C]oleic acid oxidation rate at the indicated times. Benign cells are BPH-1 and WPMY-1. *a* Benign (*n* = 6) *vs.* PCa (*n* = 9) without treatment, *t* test*—P* = 0.004. *b* Effect of 4 h treatment on oleate oxidation in PCa cells. ANOVA *P* < 0.001, *post hoc* Tukey’s: LNCaP (*P* < 0.001), VCaP (*P* < 0.001), PC3 (*P* = 0.002). **b**) Effect of etomoxir on [^14^C]palmitate oxidation. *c* Benign (*n* = 6) *vs.* PCa (*n* = 9) with no treatment, *t* test—*P* = 0.006. *d* Effect of 4-h treatment on palmitate oxidation in PCa cells. ANOVA *P* < 0.001, *post hoc* Tukey’s: LNCaP (*P* = 0.001), VCaP (*P* = 0.001), PC3 (*P* < 0.001). **c**) CPT1 isoform expression in PCa cells and BPH-1 benign line. *Post hoc* tests*,* *P ≤ 0.03 compared with BPH-1. #*P* ≤ 0.002 compared with BPH-1. **d**) CPT1A Western blot of cell lines examined. *αTUB* = tubulin loading control.

Fig. 1a shows the effect of etomoxir on the production of [^14^C]CO_2_ after incubation of the cells with [^14^C]oleate over 2 days. Note the increased oxidation rate in PCa cells (LNCaP, VCaP, and PC3) compared with the non-cancer controls (BPH-1 and WPMY-1 cells) at 0 h. VCaP cells oxidized the most oleic acid. Fig. 1b shows the effect of etomoxir on lipid oxidation rate with [^14^C]palmitate. In the absence of etomoxir treatment, LNCaP cells showed the highest rate of palmitic oxidation. Using RT-PCR, we observed that PCa cells have abundant expression of the CPT1A (liver) isoform, underscoring capacity of these PCa cells to oxidize fat; see Fig. 1c. VCaP cells showed the highest expression of CPT1A, which is parallel to the increased oleic acid oxidation capacity (Fig. 1a). Fig. 1d shows CPT1A protein expression of the cell lines examined.

**Fig. 2:**
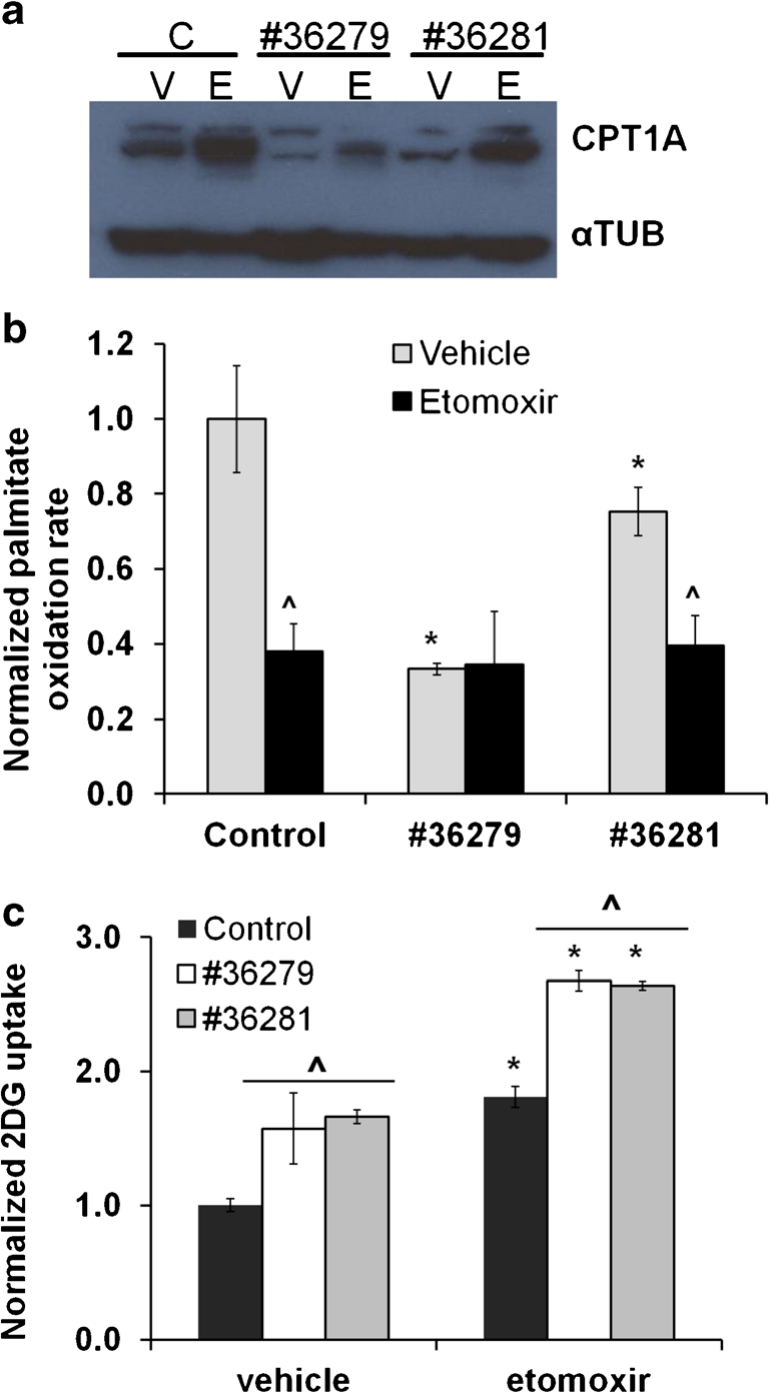
Knockdown of CPT1A results in decreased lipid oxidation and increased glucose uptake. **a**) Western blot of lysates of LNCaP CPT1A KD clones (#36279 and 36281) exposed to etomoxir for 24 h. *C* = control clone, *V* = vehicle-treated, *E* = etomoxir-treated. **b**) [^14^C]Palmitic acid oxidation rate of the control and CPT1A-KD clones, ANOVA *P* < 0.001. *Post hoc* tests, *P ≤ 0.01 compared with control clone, ^*P* ≤ 0.004 compared with vehicle treatment. **c**) Normalized 2-[^3^H] DG uptake in the LNCaP CPT1A KD clones, ANOVA *P* < 0.001. *Post hoc* tests, *P ≤ 0.001, compared with vehicle treatment, ^*P* ≤ 0.001 compared with control clone.

Since etomoxir targets the CPT-1 enzyme [19, 20], we used shRNAs to decrease CPT1A expression in LNCaP cells and mimic the pharmacological effects of etomoxir. We obtained two clones with specific knockdown (KD) of CPT1A, as shown in Fig. 2a, which surprisingly responded to 24 h etomoxir treatment by increasing CPT1A protein expression, likely reflecting a compensatory response. The 36279 and 36281 CPT1A KD clones decreased palmitate oxidation by 60 % and 25 %, respectively, when compared with control-transfected cells; see Fig. 2b. These effects were further decreased by addition of etomoxir in clone 36281 but not in 39279, suggesting maximal inhibition of beta-oxidation in the 36279 clone, which had the lowest CPT1A expression.

Since etomoxir was designed as a drug that increases glucose uptake by blocking lipid utilization [21], we examined the effects of etomoxir on glucose uptake in the LNCaP CPT1A KD clones. Both CPT1A KD clones showed significant increase of basal glucose uptake (~57 %, *P* < 0.001) compared with control the clone, plus additional increased glucose uptake after etomoxir treatment for 6 h; see Fig. 2c.

**Fig. 3:**
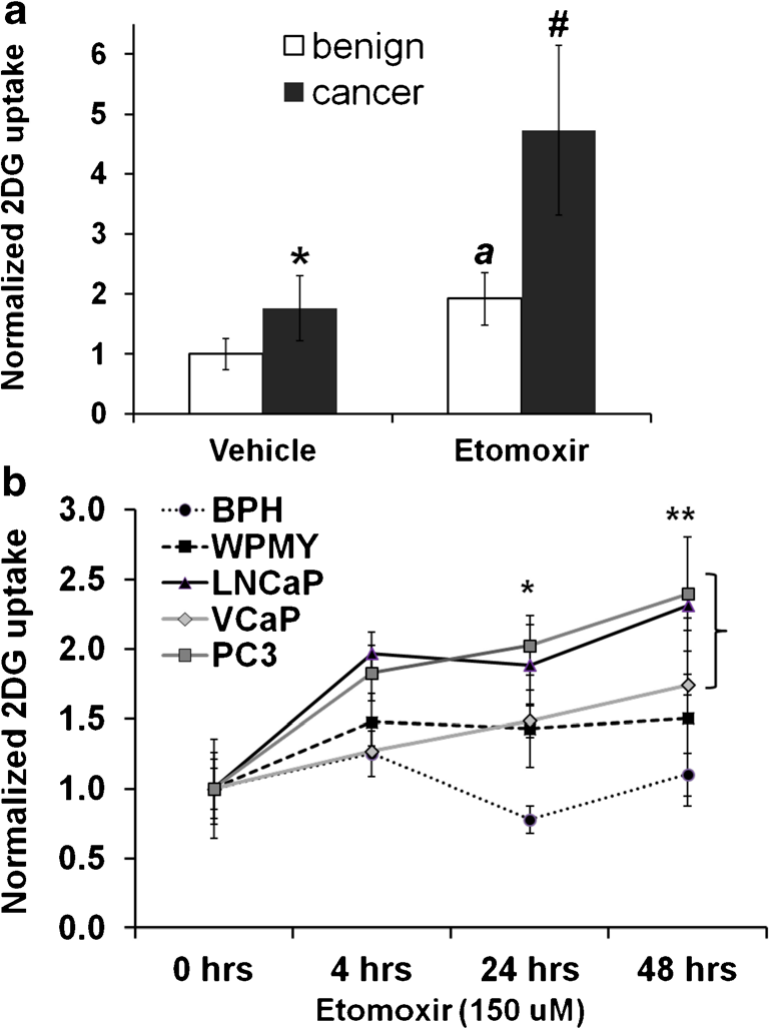
Pharmacological block in fat oxidation results in glucose uptake in PCa cells. **a**) Normalized 2-[^3^H]DG uptake in patient-matched prostate-derived primary cells. Student’s *t* test*:* *vehicle-cancer compared with vehicle-benign *P* = 0.044. *a* *P* = 0.011 compared with benign-vehicle. #*P* = 0.009 compared with vehicle-cancer. **b**) Effect of 150 μM etomoxir on the 2-[^3^H]DG uptake of prostate-derived cell lines over 48 h. *Bracket* points to PCa cells. *Etomoxir effect at 24 h between cells lines, ANOVA *P* < 0.001. *Post hoc* tests: BPH-1 *vs.* LNCaP (*P* = 0.001), PC3 (*P* < 0.001), VCaP (*P* = 0.017), WPMY-1 (*P* = 0.03). Benign WPMY-1 *vs.* PC3 (*P* = 0.043). **Etomoxir effect at 48 h between cell lines, ANOVA *P* = 0.005. *Post hoc* tests: BPH-1 *vs.* LNCaP (*P* = 0.011), PC3 (*P* = 0.007).

We next examined prostate biopsy-derived primary cells. Etomoxir treatment (6 h) resulted in significant increase in glucose uptake (1.76-fold, *P* < 0.05) in patient-derived benign prostate cells, but the effect was stronger in the adjacent tumor-derived cells (2.7-fold, *P* < 0.01); see Fig. 3a. We then extended these studies to PCa (LNCaP, VCaP, PC3) and benign (BPH-1, WPMY-1) cell lines. Overall, PCa cell lines showed progressive increase in glucose uptake compared with no treatment (0 h), ANOVA, *P* < 0.01; see Fig. 3b. Interestingly, benign BPH-1 and WPMY-1 did not show a significant increase in 2DG uptake with etomoxir; in fact, BPH-1 cells showed a slight decrease by 24 h.

In cancer cells, one of the most common avenues for glucose metabolism is its conversion into lactate, which results in media acidification. To quantify the induction of aerobic glycolysis, we obtained ^13^C-glucose spectra of VCaP cells exposed to etomoxir for 24 h (ESM Fig. 1). Table 1 shows the quantification of the extracellular concentrations of C-13-labeled glucose and lactate metabolites from the 1-[^13^C]glucose in the media of the VCaP cells. There was a twofold increase in 3-[^13^C]lactate export in the etomoxir-treated cells, concomitant with a significant increase in glucose uptake. These results strongly support the concept that the inhibition of lipid oxidation in PCa cells further induces the Warburg effect in PCa.

**Table 1:** Extracellular concentrations of C-13 labeled glucose and lactate from 1-[^13^C]glucose in VCaP cell media as calculated from C-13 NMR spectra

Vehicle-treated cells	(μM)		
	Mean	±SD	
Lactate exported	1.02	0.05	
Glucose uptake	4.77	0.56	
Glucose (extracellular)	10.39	0.73	
Etomoxir-treated cells	(μM)		
	Mean	±SD	*P* value compared with vehicle
Lactate exported	2.27	0.10	<0.001
Glucose uptake	6.46	0.71	<0.005
Glucose (extracellular)	8.57	0.69	<0.01

### Systemic Beta-Oxidation Blockade Increases [^18^F]FDG Uptake in PCa Mouse Models

**Fig. 4:**
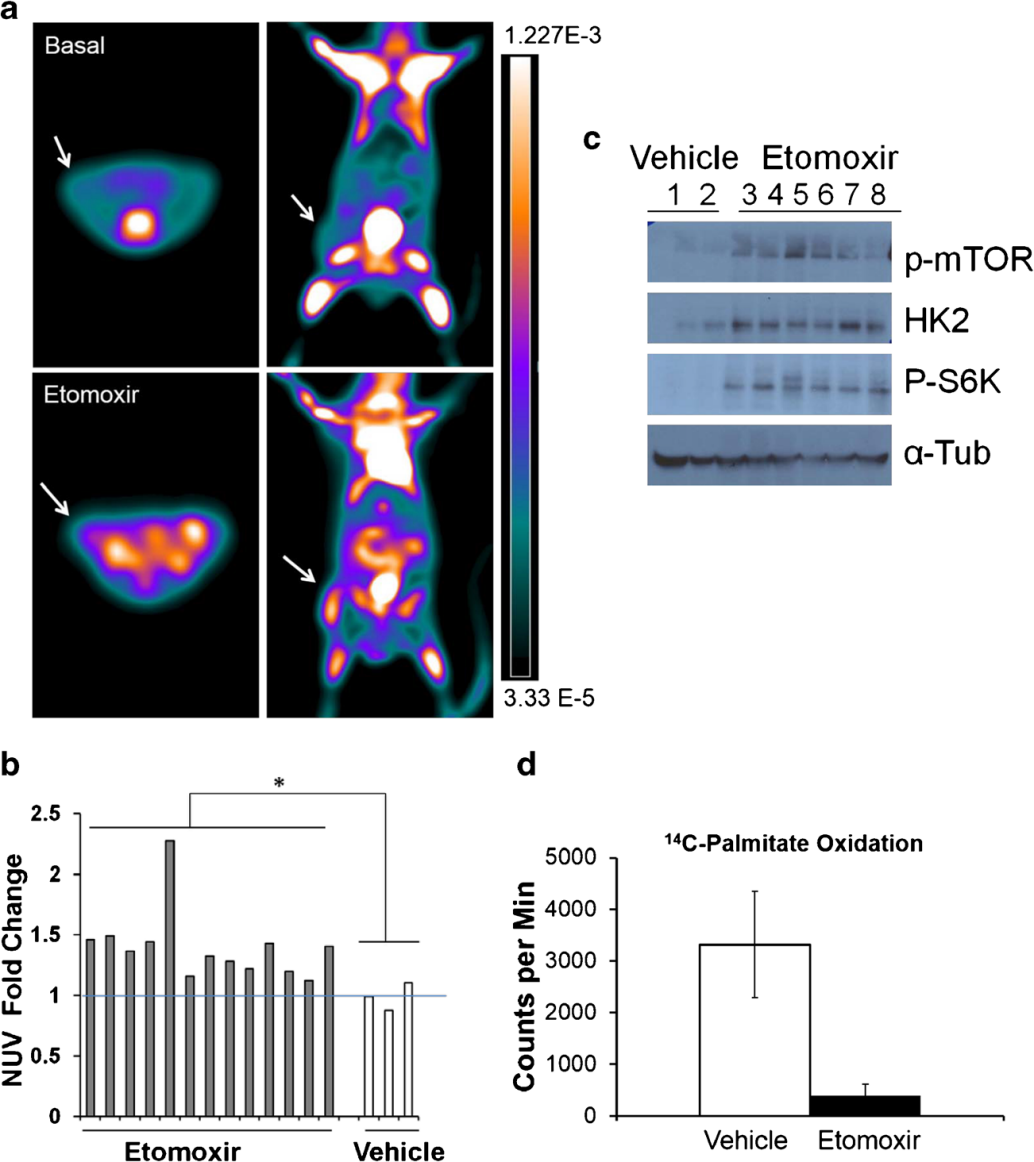
[^18^F]FDG uptake is enhanced in VCaP xenografts after systemic treatment with etomoxir. **a**) Representative axial (*left*) and coronal images of a subcutaneous xenograft mouse model before (basal, *top*) and after etomoxir injection (*bottom*). Right tumor is indicated with *white arrow*. Note the increased uptake of [^18^F]FDG in the heart with etomoxir treatment. This systemic effect was observed in all the mouse models. The scale *bar* represents signal activity as a function of radioactivity. **b**) Normalized uptake values (NUV) fold change for xenografts examined by FDG-PET, *P = 0.035 *t* test between etomoxir and water-treated tumors. **c**) Representative western blot of VCaP tumor lysates from mice treated with vehicle (water 1, 2) or etomoxir 20 mg/kg (3–8) systemically for 24 h. **d**) Radioactive counts from [^14^C]palmitate oxidation in VCaP tumor homogenates incubated *ex vivo* with water or etomoxir (150 μM) for 45 min.

To investigate if this flare of enhanced glucose uptake effect could also be happening *in vivo*, we used three mouse models of PCa: subcutaneous xenografts with VCaP cells (*n* = 16), orthotopic xenografts with PC3-Luciferase cells (*n* = 4), and genetic TRAMP mouse models (*n* = 6). All mice were treated with 20 mg/kg of etomoxir by IP injection after a basal PET scan and subjected to another PET scan after 24 h. Fig. 4a shows representative images of the before (basal) and after scans of the subcutaneous xenografts, showing increased glucose uptake (white > orange). Green color reflects the lowest uptake. Notice that the tumor (white arrow) is barely visible in the basal scan but becomes visible in 24 h with treatment. Fig. 4b shows the significant fold change difference (*P* = 0.03) in normalized glucose uptake values (NUV) between the etomoxir and water-treated subcutaneous tumors. Student’s paired *t* test of the before and after measurements for each etomoxir-treated mouse also showed a significant fold change in uptake (1.4 ± 0.3, *P* = 0.001). When we examined the NUV fold change in the mouse prostate tissue, the [^18^F]FDG uptake was minimal in the healthy prostate gland, close to the background activity. Etomoxir did not produce significant changes over basal scan (0.96 ± 0.07). Mice treated with vehicle (water) showed the same result, (1.0 ± 0.04). However, as expected, significant NUV fold changes were observed in leg muscle (1.32 ± 1.2, *P* < 0.01), subscapular brown adipose tissue or BAT (1.43 ± 1.3, *P* < 0.001) and heart (2.52 ± 0.75, *P* = 0.02) compared with basal scans. Additionally, to make the study more relevant for clinical imaging purposes, tumor-to-tissue total activity ratios were also calculated. As shown in Table 2, we observed a significant tumor/prostate ratio increase after systemic etomoxir (*P* ≤ 0.03). The tumor/muscle ratios with etomoxir were also significantly increased, reflecting the systemic effect of etomoxir in muscle tissue.

**Table 2:** Tumor-to-tissue total activity ratios in subcutaneous mouse model

	Tumor/prostate ratio (mean ± SD)		Tumor/muscle ratio (mean ± SD)		Tumor/ BAT ratio (mean ± SD)		Tumor/heart ratio (mean ± SD)	
	Pre	24-h	Pre	24-h	Pre	24-h	Pre	24-h
Etomoxir (*n* = 13)	9.06 (±6.15)	12.8^a^ (±6.68)	0.68 (±0.45)	0.85^a^ (±0.42)	0.55 (±0.35)	0.51 (±0.25)	0.64 (±0.62)	0.57 (±0.63)
Vehicle (*n* = 3)	12.67 (±4.57)	11.75 (±3.37)	0.78 (±0.36)	0.76 (±0.35)	0.56 (±0.29)	0.58 (±0.28)	0.91 (±0.49)	1.06 (±0.55)

^a^Significantly different from the basal (Pre) activity ratio (*P* ≤ 0.03, two-sided paired *t* test)

After the scans, animals were sacrificed, and tumor lysates were prepared. As expected from increased glucose metabolism, Hexokinase-2 (HK2), p-mTOR, and p-S6Kinase were increased in the etomoxir-treated tumors; see Fig. 4c. No significant changes in GLUT1 glucose transporter were observed after 24 h of etomoxir treatment; data are not shown. *Ex vivo* incubation of xenograft fragments (~20 mg) with [^14^C]palmitate and etomoxir resulted in more than tenfold decrease in [^14^C]CO_2_ production, as shown in Fig. 4d, underscoring the strong sensitivity of VCaP-based xenografts to lipid burning inhibitors.

**Fig. 5:**
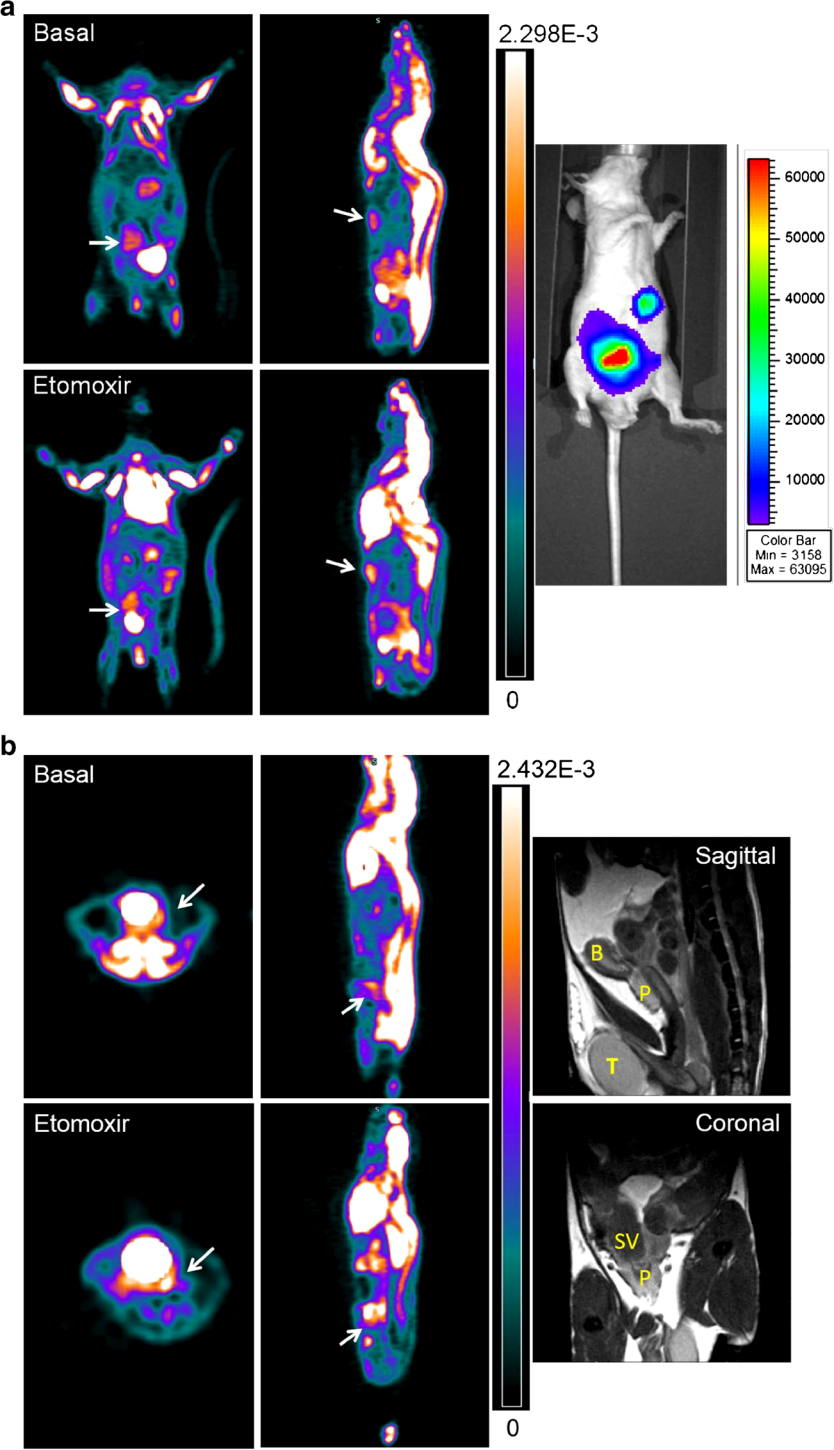
[^18^F]FDG uptake is enhanced in PC3-LUC orthotopic xenografts and TRAMP mouse models after systemic treatment with etomoxir. **a**) Coronal (*left*) and sagittal PET images of representative PC3-LUC orthotopic xenografts. Bioluminescence image on the *right* indicates where the tumor cells were growing; *red* and *blue* indicate high and low luciferase expression, respectively. In the *left panels* (coronal views), the *arrows* point to the primary tumor above the bladder (strong, circular *white* signal). In the *right panels* (sagittal views), *arrows* point to the ventral metastasis below the heart. This metastasis corresponds to upper-small signal in the adjacent bioluminescence image. **b**) Axial (*left*) and sagittal PET images of representative 24-week-old TRAMP mouse. Magnetic resonance images (sagittal and coronal) were used to anatomically visualize the increased prostate cancer growth, which extended into the seminal vesicles. *White arrows* point to tumor growth located posterior to the bladder. Bladder cannot be seen in the sagittal views, but it is visible (strong, *circular white signal*) in the axial views. MRI photographs: *B* (bladder), *P* (prostate), *SV* (seminal vesicles), *T* (testis).

Fig. 5a shows representative images of mice with orthotopic xenografts of PC3-luciferase cells. The PC3-LUC cells were surgically implanted in the right lobe of the mouse prostates. Mice in the images developed metastases in the abdomen that could be seen by bioluminescence. Only mice that showed luciferase expression were used for the PET scans. We observed a 2.7 ± 1.40-fold increase in NUV after etomoxir treatments for 24 h (*P* = 0.04). We also performed PET scans in TRAMP mice, which uniformly and spontaneously develop autochthonous (orthotopic) prostate tumors following the onset of puberty. Interestingly, like the human PCa tumors, the TRAMP tumors do not show increased expression of glucose transporters, which likely explains low [^18^F]FDG uptake [22]. Fig. 5b shows images of 24-week-old TRAMP mice, an interval at which growth of PCa is well documented [18]. The tumor behind the bladder (arrow) was significantly stronger after etomoxir treatment, and growth of prostate tissue into the seminal vesicles was observed in PET and MRI images. The increased NUV of TRAMP mice at 20 weeks was not remarkable (1.6-fold), but it was significant at 24 weeks when TRAMP tumor growth was larger (1.8 ± 0.2-fold, *P* = 0.004). Additional images of the orthotopic and TRAMP models are shown in ESM Fig. 2.

## Discussion

One important observation in our studies is the increased lipid oxidation capacity of prostate cancer cells, especially when compared with benign cells. This enhanced lipid oxidation is coupled with a decreased avidity for glucose uptake, but the mechanisms underlying this association are still debatable. Glucose transporters are not very abundant in PCa tumors, and LNCaP cells are able to grow at control rates even in the presence of 0.05 g/l of glucose [23], which may account for the low [^18^F]FDG avidity of prostate cancer. These observations suggest that PCa likely obtains additional energy from fatty acid oxidation pathways, sparing glucose use [3, 24, 25]. Furthermore, accumulating evidence suggests that other cancers also utilize fat oxidation as an important source of energy [26–28], highlighting the role of fat oxidation in overall cancer growth and survival and providing a potential link to the observation of fatty acid metabolism and PCa risk [29, 30].

This study highlights the importance of lipid catabolism in PCa cells, with potential implications for imaging of metastatic PCa. The complete ([^14^C]CO_2_) lipid oxidation results suggest that PCa cells are not only lipogenic [31] but also depend on mitochondrial lipid oxidation for energy. The results can partially be explained by abundant expression of the CPT1A enzyme in PCa cells compared with the BPH-1 cells. The association between beta oxidation and PCa cellular migration and invasion has been recently reported [32], pointing to beta-oxidation as a source of energy to sustain malignant growth.

Etomoxir is the most characterized CPT1 inhibitor [33] that forces cells to use glucose in order to maintain energy homeostasis. Thus, we also examined the effects of decreased CPT1A expression on the lipid oxidation capacity of LNCaP cells. As expected, we observed decreased fat oxidation with decreased CPT1A expression and a concomitant increase in glucose uptake, suggesting that CPT1A is mechanistically involved in the fuel switch between glucose and fat use by PCa cells. Furthermore, a significant unexpected upregulation of CPT1A expression was observed in LNCaP cells after treatment with etomoxir (Fig. 2b). We do not know the reason for this increase in protein expression, but it could be due to off-target effects of etomoxir that aim to increase fat oxidation, an effect that has been previously described in renal failure scenarios [34]. Nevertheless, this compensatory increase in CPT1A protein is likely inactive, since C-14-labeled lipid oxidation was significantly reduced with etomoxir.

The tracer studies with labeled C-13 glucose also confirmed the metabolic fate of the glucose uptake in PCa cells, as the increased [^13^C]lactate exported into the medium could only originate from the ^13^C-glucose added to the cells. Thus, the weak glycolytic potential of the prostate tumors can be enhanced by temporarily blocking their capacity to oxidize lipids.

The increased glycolysis observed with etomoxir was accompanied by increases in key glucose metabolism signaling molecules like the nutrient sensor mTOR and its downstream effector S6-kinase [35]. Activation of mTOR and S6 kinase have also been observed in mouse hearts with diminished fatty acid oxidation and compensatory catabolism of glucose [36], suggesting a reciprocal association between fat oxidation and aerobic glycolysis. Furthermore, this association between mTOR and glucose uptake has also been observed in lung cancer cells, specifically with uptake of [^18^F]FDG [37].

Hexokinase 2 (HK2), the enzyme that plays the pivotal role in the Warburg effect, was clearly increased in xenografts of mice treated with etomoxir. HK2 generates high levels of glucose-6-phosphate and initiates the glycolytic pathway, leading to lactic acid production. The technique of PET imaging is based on the activity of HK2, as it phosphorylates and traps the [^18^F]FDG inside the cells, an effect that is proportional to the tissue’s avidity for glucose [38].

To validate our *in vitro* work with etomoxir, we used three mouse models of PCa to safely enhance [^18^F]FDG uptake by the tumors using a single dose of etomoxir systemically. Recently, enhancement of [^18^F]FDG-PET in PC3 xenografts has also been reported using the antilypolytic drug acipimox, which decreases the systemic delivery of fatty acids from the adipose tissue depots [39]. The use of drugs that target CPT1 or the entry of fatty acids into the mitochondria (like etomoxir) may offer an added sensitivity, since acipimox was not effective in the more differentiated, androgen-dependent CWR22Rv1 xenografts. The VCaP cells used in our studies are slow-growing, androgen-sensitive, and possess a wild-type androgen receptor [40, 41], making them a bit closer to the nature of the indolent and [^18^F]FDG-PET “invisible” prostate carcinomas.

Additionally, glucose uptake results from our primary cancer cells also support a role for CPT1 inhibitors in enhancing the imaging of localized human tumors. However, interference of the prostate signal due to bladder proximity was problematic in the TRAMP mice. Another limitation was the increased systemic overall activity due to the overall enhancement of tracer uptake (especially in muscle, heart, and brown adipose tissue). Although muscle tissue uptake was also increased with treatment, a modest increase in the tumor/muscle ratio was observed, suggesting that overall muscle enhancement may still allow for tumor visualization in muscle. Importantly, there was no etomoxir-mediated increase in [^18^F]FDG uptake in the healthy prostate. We observed a significant increase in tumor/prostate activity ratio in the etomoxir-treated mice, underscoring the potential for FDG enhancement of local prostate tumors by systemically manipulating lipid use in tissues. However, given the increase in [^18^F]FDG uptake in heart and brown adipose tissue, metastasis in these tissues would not be observed (which have no clinical relevance since these organs do not represent the major sites for metastatic spread). Human studies will be needed to verify if the increased glucose tracer uptake also happens in the prostate gland and tumors.

Despite these drawbacks, [^18^F]FDG -PET/CT in humans is still valuable in PCa imaging. This is the case for high-grade localized disease and following known-osseous disease, as in castration-resistant PCa [42] and high post-therapeutic PSA relapse [43]. How fat-burning inhibitors like etomoxir could modify the uptake of other lipid-associated PET tracers like [^11^C]acetate and [^18^F]fluorocholine remains to be tested.

## Conclusions

Altogether, these results suggest that lipid oxidation plays a major role in glucose and lipid homeostasis in PCa cells, with potential for imaging enhancement that can also be extended to other cancers as well as distant or unexpected lesions. Although etomoxir has been used clinically, it is not FDA-approved. Our metabolic findings open the door to rational clinical designs to explore metabolite-based PET imaging for prostate cancer. Other fat oxidation-related drugs that are clinically approved for heart failure (ranolazine, trimetazidine) may offer new imaging avenues to be tested.

## Electronic supplementary material

Below is the link to the electronic supplementary material.

ESM 1 (PDF 1446 kb)
