# Supplementary material for: Inhibition of Lipid Oxidation Increases Glucose Metabolism and Enhances 2-Deoxy-2-[18F]Fluoro-d-Glucose Uptake in Prostate Cancer Mouse Xenografts
Source: Mol Imaging Biol. 2015 Jan 6;17(4):529–38. doi: 10.1007/s11307-014-0814-4 (PMC4493937; doi:10.1007/s11307-014-0814-4)
Supplement: Supplementary file 1 — (PDF 1446 kb) [file 11307_2014_814_MOESM1_ESM.pdf]

Supplementary Material

Inhibition of lipid oxidation increases glucose metabolism and enhances 2-deoxy-2-[^{18}F]-fluoro-D-glucose uptake in prostate cancer mouse xenografts.

Journal: Molecular Imaging & Biology

Isabel R. Schlaepfer*, L. Michael Glodé, Carolyn A. Hitz, Colton T. Pac, Kristen E. Boyle,

Paul Maroni, Gagan Deep, Rajesh Agarwal, Scott M. Lucia, Scott D. Cramer,

Natalie J. Serkova and Robert H. Eckel

*Corresponding Author

Isabel R. Schlaepfer Ph.D.
Assistant Professor
University of Colorado School of Medicine | Division of Medical Oncology
Genitourinary Cancer Program
MS 8117 | 12801 E. 17th Ave, Room L18-8101D | Aurora, CO 80045
ph: 303-724-8867
isabel.schlaepfer@ucdenver.edu

**Suppl. Table 1: Primer sequences for human CPT-1 expression**		
**Sequence (5'—>3')**	**Length**	**Name**
TGGTGCTCAAGTCATGGTGG	20	CPT1B-F
TGCCTGCACGTCTGTATTCT	20	CPT B-R
TTGGACCGGTTGCTGATGAC	20	CPT 1A-F
GGTGCCTTCCAAAGCGATGA	20	CPT1A-R
GCGCAAAAAGGCAGCTGAA	19	CPT 1C-F
ACACACCGGTGAGAAAGTCA	20	CPT1C-R
AGGCTATCCAGCGTACTCCAAAGA	24	B2M-F
TCGGATGGATGAAACCCAGACACA	24	B2M-R

B2M : Beta-2-microglobulin

Supplementary figures

Supplementary Figure 1

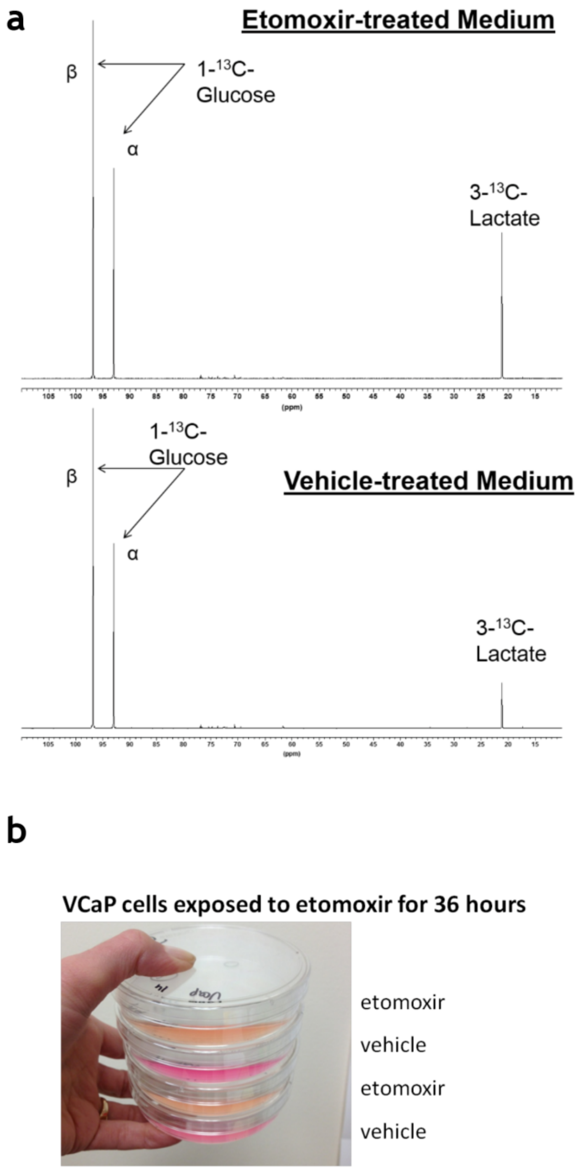

Supplementary figure 1: NMR trace of VCaP cells
a) Representative ^{13}C-Magnetic resonance spectra on cell media of VCaP cells treated with vehicle (top panel) or etomoxir (bottom) for 24 hours. The two epimers of 1-^{13}C-glucose are indicated (α and β).
b) Effect of etomoxir on the media acification of VCaP cells. The yellow color of the media indicates lower pH characteristic of increased lactate exported from the cells.

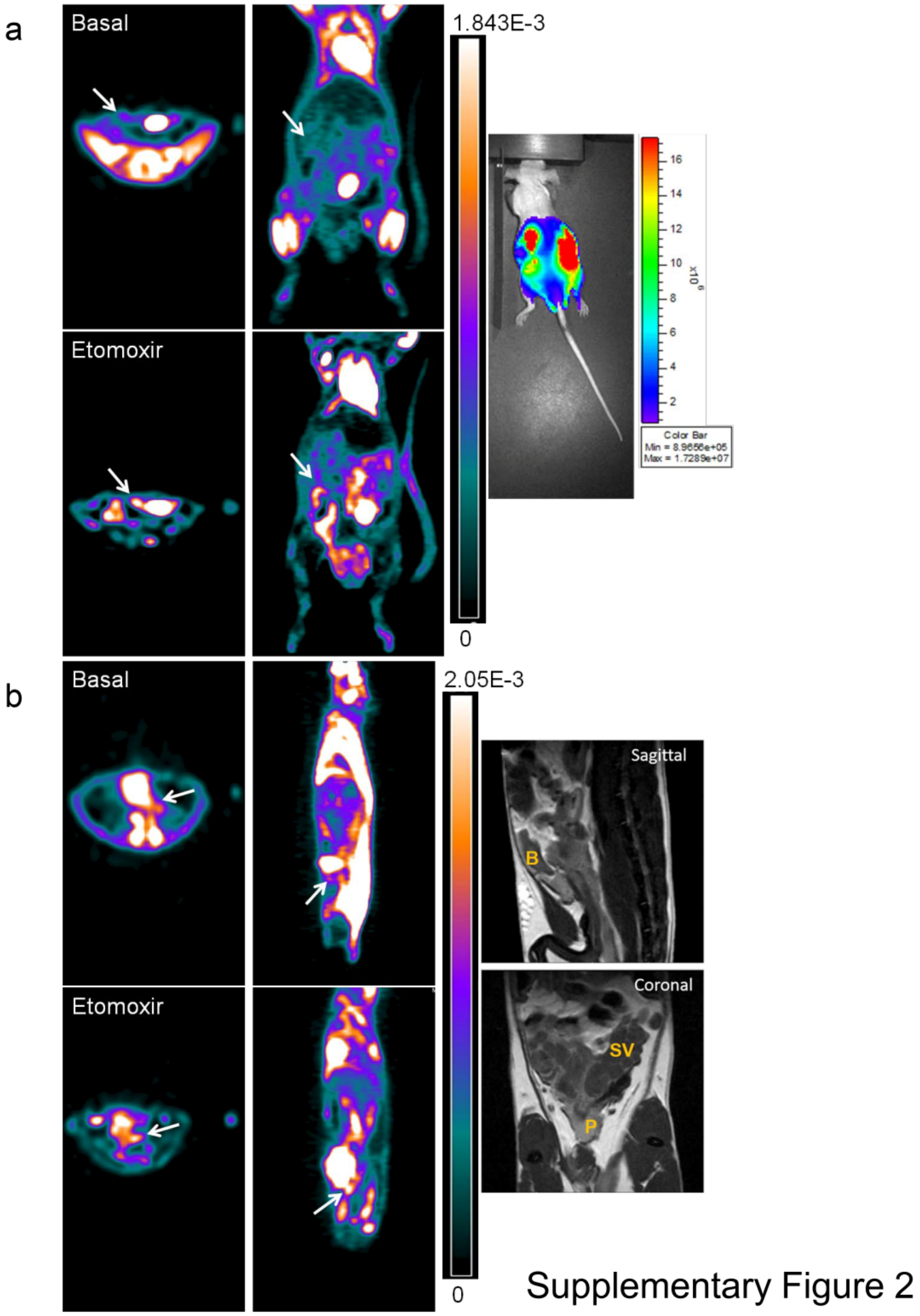

Supplementary figure 2: Additional PET scan images

a) Axial and Coronal images of PC3-LUC orthotopic xenograft model before and after etomoxir. Note metastatic tumor growth on right side (*arrow*). Tail of mouse is left side. Cells were injected on the right anterior lobe of prostate. Implanted primary tumor is indicated by arrow on the axial image. Note metastasis signal above bladder on the coronal section after etomoxir treatment.

b) Axial and sagittal images of 24-wk old TRAMP mouse before and after etomoxir. Arrow points to prostate area, inferior/posterior to bladder (bladder is the strong white round signal in the sagittal images). MRI images show prostate growth. B=bladder, SV=seminal vesicle, p=prostate. Note prostate tissue extending into seminal vesicles.
